# Ok Google: Using virtual assistants for data collection in psychological and behavioral research

**DOI:** 10.3758/s13428-021-01629-y

**Published:** 2021-09-10

**Authors:** Philipp Sprengholz, Cornelia Betsch

**Affiliations:** 1 Media and Communication Science, University of Erfurt, Erfurt, Germany; 2 Center for Empirical Research in Economics and Behavioral Sciences, University of Erfurt, Erfurt, Germany

**Keywords:** Virtual assistant, Data collection

## Abstract

Because of the increasing popularity of voice-controlled virtual assistants, such as Amazon’s Alexa and Google Assistant, they should be considered a new medium for psychological and behavioral research. We developed Survey Mate, an extension of Google Assistant, and conducted two studies to analyze the reliability and validity of data collected through this medium. In the first study, we assessed validated procrastination and shyness scales as well as social desirability indicators for both the virtual assistant and an online questionnaire. The results revealed comparable internal consistency and construct and criterion validity. In the second study, five social psychological experiments, which have been successfully replicated by the Many Labs projects, were successfully reproduced using a virtual assistant for data collection. Comparable effects were observed for users of both smartphones and smart speakers. Our findings point to the applicability of virtual assistants in data collection independent of the device used. While we identify some limitations, including data privacy concerns and a tendency toward more socially desirable responses, we found that virtual assistants could allow the recruitment of participants who are hard to reach with established data collection techniques, such as people with visual impairment, dyslexia, or lower education. This new medium could also be suitable for recruiting samples from non-Western countries because of its wide availability and easily adaptable language settings. It could also support an increase in the generalizability of theories in the future.

## Introduction

Dave: Open the pod bay doors, HAL.

HAL: I’m sorry, Dave. I’m afraid I can’t do that.

Dave: What’s the problem?

HAL: I think you know what the problem is just as well as I do.

Dave: What are you talking about, HAL?

HAL: This mission is too important for me to allow you to jeopardize it.

When Dave Bowman and other astronauts are sent on a space mission in Stanley Kubrick’s movie *2001 – A Space Odyssey*, their ship is equipped with HAL, a voice-controlled computer system. As HAL begins to malfunction, the astronauts decide to shut it down. To avoid disconnection, the system starts disobeying orders and killing the crew, which leads to a showdown between the men and the machine. While fostering suspicion against intelligent machines, the movie also aroused dreams about a future in which interactions with devices would require nothing but our voice. With ever-increasing computer processing power and major advances in machine learning and natural language processing, this dream has finally been realized (Hirschberg & Manning, 2015). Among the most significant innovations that employ voice technology are virtual assistants—software agents such as Apple’s Siri, Amazon’s Alexa, and Google Assistant—that perform tasks or services based on user commands or questions. This includes searching for information, managing to-do lists and calendars, playing music, or controlling home automation devices (Ammari et al., 2019; Hoy, 2018). In recent years, an increasing number of people have started using virtual assistants on both mobile phones and home appliances, including smart speakers and smart televisions. As of 2019, more than three billion virtual assistants were available on devices worldwide. Forecasts suggest that they will overtake the world population by 2023 (Juniper Research, 2019). In the United States, the total number of smart speakers increased by 135% between the end of 2017 and the end of 2019, and 60 million Americans own an average of more than two speakers (NPR & Edison Research, 2020). While the United States is the most advanced market for virtual assistant adoption, a study by Pérez García et al. (2018) revealed that in other countries, such as the United Kingdom, Spain, Germany, Brazil, and Chile, between one-quarter and one-third of the population is using virtual assistants, more than half of whom are using them on a daily basis.

The functionality of virtual assistants is not limited to built-in capabilities. Programming interfaces allow third-party developers to create additional “skills” to extend the functionality of assistants, with the number of available skills depending on the country and platform. For instance, in the United States, Alexa users can choose from more than 60,000 skills, including extensions for playing quizzes, ordering pizza, or reading bedtime stories. Researchers have also started developing and evaluating skills for educational and healthcare purposes. For instance, virtual assistant skills have been shown to foster second-language learning (Dizon, 2017) or support the collection of medical data from patients with cardiovascular disease during patient registration at hospitals (Jadczyk et al., 2019). While virtual assistants are still some distance away from assuming HAL’s capabilities, continuous improvements in artificial intelligence are paving the way for new applications.

Because of their increasing popularity, virtual assistants should be considered a novel method of data collection in social and psychological research as they could be employed to survey individuals about their thoughts, feelings, and behaviors. Compared to established data collection techniques, such as paper and online questionnaires, survey skills could reach a wider range of people for the following two reasons:

1. Availability: While the extensive use of smart home devices is limited to industrialized countries, smartphones are common across the globe, even in low- and middle-income countries (Pew Research Center, 2019). As virtual assistants come preinstalled and new skills are set up within seconds, psychological studies could be made available to millions of users.
2. Accessibility: Common questionnaires require manual input. Participants have to read instructions and use a pencil or keyboard to answer questions and complete experimental tasks. This process is not necessary when interacting with a virtual assistant, which can read instructions aloud, and participants can answer using only their voice. This approach allows easier integration into everyday life. People can participate in a psychological study hands-free while waiting for the bus or ironing the laundry. It also fosters the participation of persons for whom reading or writing might be challenging (Davie & Hilber, 2018).

Despite its potential, the reliability and validity of data collected through virtual assistants have never been evaluated. The expected advantages could be accompanied by adverse effects that limit data quality. For instance, the hands-free nature of communication could cause decreased or unsteady attention, and technical issues could hamper the correct identification of a user’s utterances or impact the usability and acceptance of the medium. Since online questionnaires and experiments can be considered standards of data collection (Sassenberg & Ditrich, 2019), we decided to conduct two studies to investigate whether the quality of the data collected through virtual assistants would be comparable to data collected using regular online survey tools. To collect data for both studies, we developed Survey Mate, a Google Assistant skill capable of running surveys.

## How Survey Mate works

**Fig. 1:**
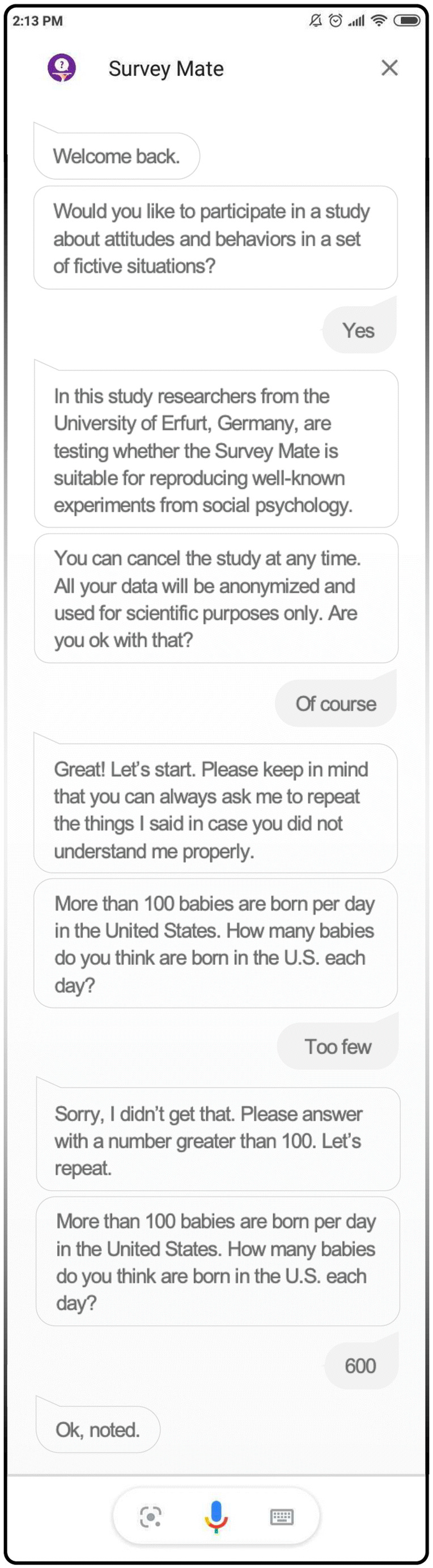
Example of a Survey Mate study running on a smartphone. *Note.* Survey Mate recognizes fuzzy and synonym input (e.g., “yes,” “of course,” or “I do”). All instructions and questions are read aloud by the voice assistant, and users can answer using their voice and/or keyboard (if available). A backend system registers and stores all answers for later analysis and ensures that users can only participate once in the same survey

Surveys intended for virtual assistants are programmed on a backend server and rolled out to Survey Mate once it starts. The skill explains how the collected data will be used and asks for consent before presenting the survey questions (Fig. 1). Different types of questions can be programmed, ranging from simple yes/no questions (e.g., Do you have children?) to closed questions about numbers (e.g., How old are you?), frequencies (e.g., How often do you feel alone?), or the level of agreement (e.g., How much do you blame yourself for your past behaviors?). For each type, a set of possible answers and synonyms is defined; for instance, frequency questions can be answered on five-point scales (including the answer categories “never,” “rarely,” “sometimes,” “often,” and “very often”) as well as allow synonyms (e.g., treating answers such as “seldom” or “barely” as “rarely”). If a user’s answer cannot be matched to one of the answer categories, Survey Mate informs the user about answer options and repeats the question. The order of the questions can be randomized, and users can be assigned to different groups (e.g., experimental conditions) with different instructions or questions. Survey Mate can be installed on all Google Assistant-enabled devices, including phones, tablets, and smart speakers. Depending on the device, questions are read aloud and/or displayed, and users can answer using their voice and/or keyboard. All input (e.g., a user utters “yes I do” after being asked whether she wants to participate in a new survey) is sent to Google’s dialog management system, where it is translated into a specific request (e.g., the system detects that a new survey has to be started). The request is then forwarded to the backend server where all surveys, questions, and answers are stored. A text response (e.g., a consent question) is generated there and sent back to Google, where it is transformed into speech files that the assistant can display and read aloud before waiting for the next user input. The current version of Survey Mate can be installed from the Google Assistant directory (https://assistant.google.com/services/a/uid/000000758eed8be0).

## Overview

For virtual assistants to be considered a suitable tool for data collection, multiple requirements should be met. Two studies were conducted to investigate the preconditions. In the first study, we compared the quality of personality data collected with both Survey Mate and online questionnaires. In the second study, we reproduced a set of classic and contemporary experiments from social psychology using Survey Mate. We examined the following four research questions:

1. Are the internal consistencies of psychological scales assessed with Survey Mate comparable to (a) those obtained from collecting data using a regular online questionnaire and (b) those obtained from reliability figures reported by previous research?
2. Are the construct and criterion validity (e.g., correlation of scales with behavioral intentions and intercorrelation between scales) comparable for data collected using Survey Mate and an online questionnaire?
3. Are the mean results obtained with Survey Mate comparable to the results obtained from an online questionnaire (e.g., means of psychological scales and experimental results)?
4. Is using the virtual assistant perceived as more difficult or enjoyable compared to a questionnaire?

## Study 1

In the first study, we compared the reliability and validity of the personality scales, as assessed with Survey Mate or by means of an online questionnaire. Although it is preferable to use the validated original statements of the scales, working with questions more closely resembles natural conversation and may better fit the dialog structure provided by the virtual assistant (Moore & Arar, 2019). Therefore, we also assessed whether responding to questions instead of agreeing with statements would affect the reliability and validity of the results. Since previous research has shown that many virtual assistant users are concerned about data privacy (Ammari et al., 2019), we further explored whether participants using Survey Mate or an online questionnaire differed in their willingness to answer sensitive questions and their trust in our promise to protect their data and use it exclusively for scientific purposes.

### Method

#### Design and procedure

The study implemented a 2 (medium: assistant vs. questionnaire) × 2 (phrasing: statements vs. questions) factorial between-subjects design. The participants were randomly assigned to the media and phrasing conditions. In the first part of the study, three established personality scales were assessed: the Irrational Procrastination Scale (IPS) (Steel, 2010), the Revised Cheek and Buss Shyness Scale (RCBS) (Cheek, 1983), and the Social Desirability – Gamma Short Scale (KSE-G), which comprises two subscales: Exaggerating Positive Qualities (PQ+) and Minimizing Negative Qualities (NQ−) (Nießen et al., 2019). In the second part of the study, all the participants were asked to complete a short online questionnaire about their experiences during the first part, their intended behavior in procrastination and shyness-related scenarios, and their satisfaction with life using the Satisfaction with Life Scale (SWLS) (Diener et al., 1985).

#### Participants

Since we were interested in the medium-sized effects of the potential differences between the conditions (Cohen’s *f* = 0.25), the sample size was calculated to 210 (based on α = .05 and a statistical test power of 1-β > .95). The data collection occurred in November 2019. A total of 240 Americans with a Google Assistant-enabled phone or smart home device were recruited online using Amazon Mechanical Turk, a crowdsourcing platform known for inexpensive, rapid collection of high-quality data (Buhrmester et al., 2011). The participants were between 20 and 69 years old (*M* = 33.77, *SD* = 9.77); 65.8% were male; and 32.5% were female. More than two-thirds had a university degree, and the average household income was comparable to the US median (see online supplement). Among the 120 subjects in the virtual assistant condition, 79% used a smartphone, 12% a smart home speaker, and the remainder a tablet or television to participate in the first part of the study. Of the subjects, 38% listened to Survey Mate’s instructions and questions, while 28% read them. Approximately one-third of the participants indicated that they had both listened to and read the instructions. In addition, 42% used only their voice to answer questions, 44% used only a keyboard, and 14% used a combination.

The participants received a fixed compensation of 0.60 USD and an additional payment of 0.60 USD when they were assigned to the virtual assistant condition as it was anticipated that setting up Survey Mate and answering its questions would consume considerably more time than completing the online questionnaire. The participants in the questionnaire condition spent a mean time of 2 min and 41.0 s (*SD* = 57.9 s) answering the items in the first part of the study, while those in the virtual assistant condition spent 4 min and 27.4 s on average (*SD* = 1 min and 1.6 s), excluding time spent setting up Survey Mate before the first use.

#### Materials and measures

In the first part of the study, the procrastination, shyness, and social desirability ratings were assessed based on the assigned media and phrasing conditions. All other measures were collected in the second part, using the same online questionnaire for all participants. The order of both the scales and their items was randomized. This procedure, content, and randomization applied to all conditions.

##### Medium manipulation

The participants were randomly assigned to one of the two media conditions and asked to answer a short survey using either Survey Mate (assistant condition) or the online questionnaire (questionnaire condition).

##### Phrasing manipulation

The presentation of the items depended on the phrasing condition to which the participants were randomly assigned. In the statement condition, the items were presented as statements, and no changes to the original texts were made. The participants rated their agreement on a five-point scale ranging from strongly disagree to strongly agree (example items: “I delay tasks beyond what is reasonable”; “I do not find it hard to talk to strangers”; “I always remain objective and stick to the facts”). In the question condition, the original items were changed along with the answer scale. First, the items were rephrased as questions (for example, the IPS item “I delay tasks beyond what is reasonable” was changed to “Do you delay tasks beyond what is reasonable?”). Second, negated items were reversed (for example, the RCBS item “I do not find it hard to talk to strangers” was changed to “Do you find it hard to talk to strangers?”). Third, using a five-point scale ranging from “never or very rarely” to “very often,” the participants rated the frequency with which the addressed feelings, thoughts, and behaviors occurred.

##### Procrastination, shyness, and social desirability

The IPS (nine items), RCBS (13 items), and KSE-G (six items) were assessed using the previously mentioned five-point scale.

##### Difficulty and enjoyment

With two items, the participants were asked how difficult they considered the first part of the study and how much they enjoyed responding (five-point scale ranging from “not at all” to “very much”). As an indirect measure of difficulty, we also registered how often Survey Mate had to repeat items because it could not understand or classify the users’ answers.

##### Behavioral intention in procrastination and shyness scenarios

The participants read two fictitious scenarios created based on IPS and RCBS items (see online supplement). In the first scenario, they were to imagine being assigned a job that required some hours to complete. They were to rate how likely they were to start working on it immediately, given that they were free for the next hours. In the second scenario, they were to imagine being at a party where they did not know anybody. They were to rate how likely they were to start conversations with strangers. For both scenarios, a ten-point scale was utilized, ranging from “very unlikely” to “very likely.”

##### Satisfaction with life

The SWLS (five items; example item: “In most ways, my life is close to my ideal”) was assessed using a seven-point scale ranging from “strongly disagree” to “strongly agree.”

##### Willingness to share sensitive information

In the first part of the study, the participants were asked how likely they were to share information about their salary and income, sex life, voting decisions, receipt of public assistance, dishonesty, drug use, crimes, victimization, and infections and diseases when using the medium. Each topic was assessed on a four-point scale, including the following options: (1) would refuse to answer, even if not being paid for participation afterwards; (2) would refuse to answer but only if still getting paid; (3) would answer but would not like it; and (4) would like to answer.

##### Trust in privacy

At the beginning of the study, the participants were informed that their data would be anonymized and employed exclusively for scientific purposes. After finishing the first part, they were asked how much they trusted this statement. A five-point scale was applied, ranging from “not at all” to “very much.”

### Results

To answer R1 regarding reliability, we compared the internal consistencies of the scales assessed in the first part (a) between Survey Mate and the questionnaires and (b) with the reliability figures reported in previous research. For the English versions of the scales, Cronbach’s alphas of .91 for the IPS (Steel, 2010), .86 for the RCBS (Hopko et al., 2005), and .65 and .79 for the PQ+ and NQ– subscales, respectively, of the KSE-G (Nießen et al., 2019) were reported. To answer R2 regarding construct and criterion validity, correlations between the IPS/RCBS and SWLS, as well as the intended behavior in the procrastination/shyness scenarios, were assessed and compared across the media conditions.

Previously, correlations were identified between the IPS and SWLS of *r* = – .41 (Svartdal & Steel, 2017) and the RCBS and SWLS of *r* = – .31(Liu et al., 2018). To answer R3, we compared the means of the IPS, RCBS, and KSE-G ratings between the media conditions, and to answer R4, we compared the subjective experience of taking part in the study across the media conditions. In all the analyses, we assessed the effect of the phrasing condition on all criteria and tested for statistically significant differences. While non-significant findings should not be mistaken for equivalence of conditions, we interpreted them as an indicator of comparability. All data analyses were performed in R. All codes and results are provided in the online supplement.

#### Reliability

Cronbach’s alpha was calculated to estimate the internal consistency of the IPS, RCBS, and KSE-G assessments. Acceptable to excellent consistencies were obtained for all scales and conditions (Table 1). For the IPS and the PQ+ and NQ– subscales of the KSE-G, no statistically significant differences between the media and phrasing conditions were found. However, for the RCBS, reliability estimates could not be considered equal, χ^2^ (3, *N* = 240) = 23.55, *p* < .001, revealing a significantly lower (but still high) scale consistency when using statement phrasing in the assistant condition. Overall, the reliability requirements of research question R1 was met.

**Table 1:** Descriptive statistics and correlations for the procrastination (IPS), shyness (RCBS), and social desirability (KSE-G) scales

	Medium	Phrasing	*n*	*M*	*SD*	α	*r*_SWLS_	*r*_scenario_
Procrastination (IPS)	Questionnaire	Statement	60	2.75	0.97	.91	– .48	– .49
		Question	60	2.88	0.83	.90	– .33	– .36
	Assistant	Statement	60	2.43	0.82	.90	– .36	– .52
		Question	60	2.69	0.84	.89	– .38	– .50
Shyness (RCBS)	Questionnaire	Statement	60	2.93	1.04	.94	– .43	– .71
		Question	60	2.99	1.13	.97	– .40	– .50
	Assistant	Statement	60	2.56	0.77	.88	– .33	– .71
		Question	60	2.68	0.97	.94	– .56	– .67
Exaggerating positive qualities (KSE-G PQ+)	Questionnaire	Statement	60	3.53	0.87	.76		
		Question	60	3.65	0.85	.76		
	Assistant	Statement	60	3.91	0.80	.80		
		Question	60	3.99	0.75	.82		
Minimizing negative qualities (KSE-G NQ–)	Questionnaire	Statement	60	2.60	1.10	.73		
		Question	60	2.55	1.10	.78		
	Assistant	Statement	60	2.04	0.81	.80		
		Question	60	2.21	0.82	.79		

*Note:* For the calculation of *r*_scenario_, different scenarios were applied for the IPS (intended behavior in a fictitious job scenario) and RCBS (intended behavior in a fictitious party scenario) scales. For the KSE-G, no reference data are available from previous research. All correlations were significant with *p* < .05

#### Construct validity

Table 1 shows moderate to strong negative correlations of the IPS and RCBS with the SWLS (*M* = 4.61, *SD* = 1.67, Cronbach’s α = .94). As expected, higher procrastination and shyness scores were related to lower satisfaction with life. For the IPS, the correlations ranged between – .33 and – .48 and were comparable to the figure (– .41) reported by Svartdal and Steel (2017). For the RCBS, correlations with the SWLS ranged between – .33 and – .56, which were stronger than the reference (– .31) reported by Liu et al. (2018). No evidence of the differences between the media and phrasing conditions was observed: *z* = 1.548, *p* = 0.122. While this may have been due to the sample size, the results indicate construct validity (R2).

#### Criterion validity

Table 1 shows moderate to strong correlations between the IPS ratings and the intention to start working in the fictitious job scenario. A comparison between the weakest and strongest correlation coefficients using the R package *cocor* (Diedenhofen & Musch, 2016) revealed no evidence of differences between the media and phrasing conditions, *z* = 1.065, *p* = .287. For the RCBS, strong correlations with the intention to start conversations with strangers at a fictitious party were obtained for all conditions. There were no significant differences between the smallest and largest coefficients, *z* = 1.804, *p* = .071. While the null finding may have been due to the sample size, the results indicate criterion validity, as required by R2.

#### Comparison of means

Two-way ANOVAs with media and phrasing as factors and scale means as dependent variables showed that the mean values for the IPS, RCBS, and KSE-G NQ– were slightly lower in the assistant condition, while the mean of the KSE-G PQ+ subscale was slightly higher (Table 1; for detailed results, refer to the online supplement). However, only the difference in exaggerating positive qualities was found to be significant, *F*(1, 236) = 5.150, *p* = .024. Moreover, there were no significant main effects for phrasing and no significant interaction effects of media and phrasing on the IPS, RCBS, and KSE-G ratings.

#### Difficulty of use and enjoyment

**Fig. 2:**
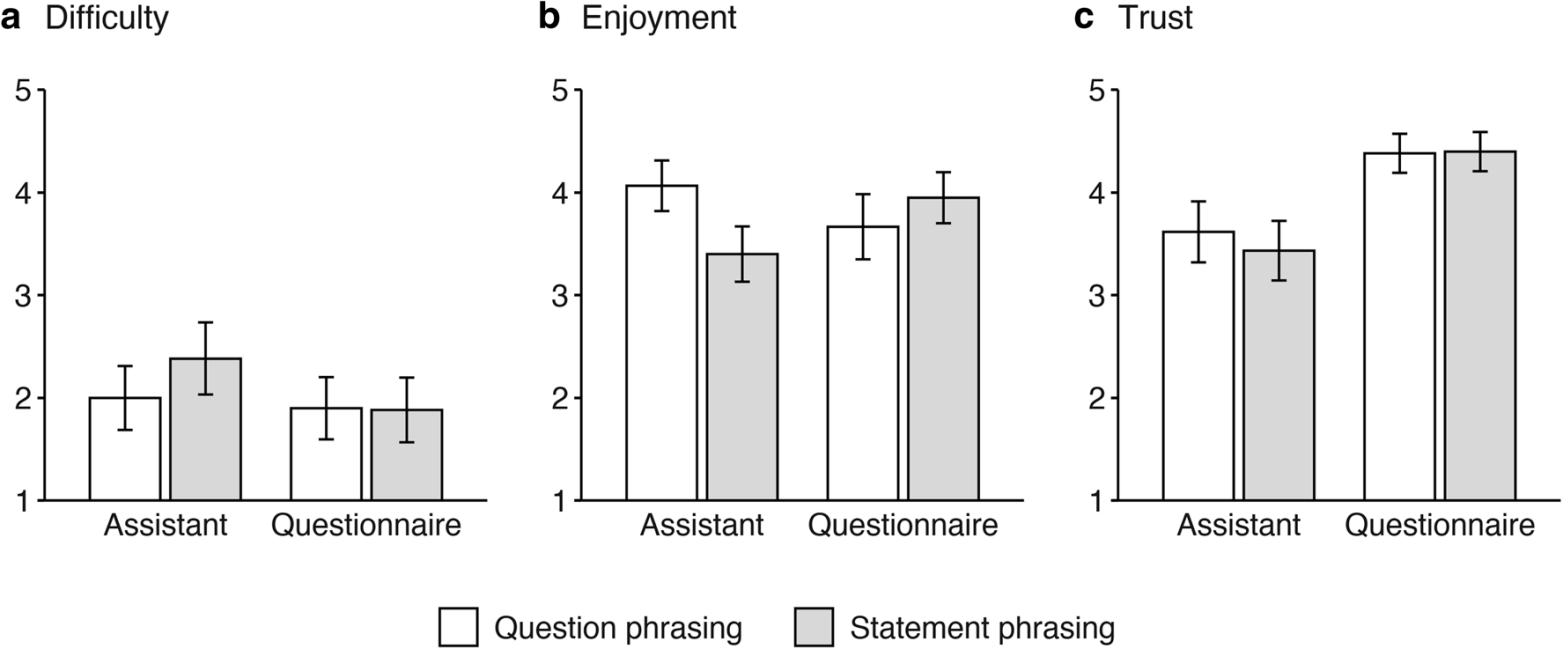
Reported difficulty, enjoyment, and trust in the first part of Study 1. *Note.* While difficulty ratings (**a**) were comparable between conditions, enjoyment and trust were not. For assistant users, receiving questions was enjoyed more than receiving statements (**b**). Furthermore, assistant users indicated lower trust in the promise that their data would be anonymized and employed only for scientific purposes (**c**). Error bars indicate 95% confidence intervals

The participants reported low to medium difficulties in completing the first part of the study. Compared with all other conditions, this seemed to be slightly more difficult for the assistant users in the statement phrasing condition (Fig. 2a). However, an ANOVA revealed no significant main or interaction effects for the media and phrasing conditions on perceived difficulty (for details, refer to the online supplement). This result is consistent with the low number of repetitions in the assistant conditions: on average, only 2.4% (0.68 items) of the 28 IPS, RCBS, and KSE-G items had to be repeated by Survey Mate. Furthermore, no significant effects of phrasing on the number of repetitions were observed, *|t| < 1*.

Enjoyment was rated medium to high (Fig. 2b). While no main effects for media or phrasing on the level of enjoyment could be detected by the ANOVA, an interaction effect emerged, *F*(1, 236) = 13.537, *p* < .001. A post hoc Tukey test showed that assistant users enjoyed the study much more when question phrasing was utilized instead of statement phrasing, *p* = .004. All other differences were not significant (see online supplement). With regard to R4, the results indicate that virtual assistant surveys can be designed to be perceived as easy and enjoyable as questionnaires.

#### Trust and willingness to share sensitive information

Trust in the promise that the participants’ data would be anonymized and utilized only for scientific purposes differed between the media conditions (Fig. 2c). An ANOVA investigating the effects of both media and phrasing on trust revealed that the assistant users had significantly lower trust in the protection of their data, *F*(1, 236) = 48.9, *p* < .001, all other *Fs* < 1. Interestingly, while there was lower trust in the assistant than the questionnaire survey regarding data protection, the willingness to share sensitive information did not differ between the two media. After comparing the proportion of participants in the assistant and questionnaire conditions who would have liked to share information about salary and income (53 vs. 51%), sex life (44 vs. 46%), voting decisions (63 vs. 58%), the receipt of public assistance (53 vs. 52%), dishonesty (50 vs. 51%), drug use (58% both), crimes (46 vs. 48%), victimization (45 vs. 43%), and infections and diseases (45 vs. 39%), no significant differences were observed, all *|t|* < 1.

### Discussion

The results indicate that virtual assistants are broadly capable of collecting reliable and valid data, making them comparable to online questionnaires. The four research questions can be answered as follows: Reliability estimates for the IPS, RCBS, and KSE-G were high (R1), and the expected negative correlations of the IPS and RCBS with the SWLS and intentions in two fictitious scenarios were obtained, thereby indicating construct and criterion validity (R2). When examining the scale means, comparable results were obtained for both media in most cases (R3). However, the significantly higher ratings of the assistant users on the PQ+ subscale of the KSE-G and the lower but non-significant ratings on the IPS, RCBS, and NQ– indicate a trend toward more socially desirable responses in assistant versus questionnaire surveys. Regarding the psychometric criteria, the phrasing of items did not seem to play an important role. However, answering questions instead of rating statements was enjoyed much more by the virtual assistant users. While not significant, the average perceived difficulty was also higher when the virtual assistant users were presented with statements instead of questions. We conclude that when using a digital assistant for data collection, rephrasing existing statement-based scales into batteries of questions is advisable to ensure that the new medium is enjoyed and accepted (R4).

In conclusion, Survey Mate satisfied the requirements for a reasonable use of virtual assistants for the collection of high-quality psychological data. However, significantly lower trust in the virtual assistant than the online questionnaire regarding the protection of personal data may limit the range of applications. Interestingly, the willingness to share sensitive information, such as details about income and salary, crimes committed, or one’s sex life, did not differ between the assistant and questionnaire users. This finding is consistent with that of previous research showing that virtual assistant users often do not articulate a coherent viewpoint when asked about their privacy concerns (Ammari et al., 2019). In many cases, this shortcoming is attributed to the notion that users do not have a clear understanding of how data are processed and what information is shared with other parties.

Importantly, about half of the participants in the virtual assistant condition used a smartphone keyboard, not their voice, to answer the survey items. Therefore, we decided to explore potential differences between both input methods in a second study.

## Study 2

While Study 1 indicated that virtual assistants were capable of collecting reliable and valid survey data, Study 2 focused on their capability in term of conducting experiments characterized by longer scenario descriptions. We aimed to reproduce five classic and contemporary experiments from social psychology that had already been successfully replicated by the Many Labs projects (Klein et al., 2014, 2018). We assumed that the effects would also be identified when conducting the experiments using a virtual assistant such as Survey Mate. Furthermore, we explored whether the type of device (smartphone vs. smart speaker) would influence the results. The study was preregistered (http://aspredicted.org/j2er6.pdf).

### Method

#### Design and procedure

For each of the five experiments, a 2 (depending on the experiment: condition 1 vs. condition 2) × 2 (device: smartphone vs. smart speaker) factorial between-subjects design was implemented. Each participant took part in all five experiments. The order of the experiments and assignment to the experiment conditions were randomized. However, the device was not randomized; they were chosen by the participants since we assumed that some of them did not own both a Google Assistant-enabled smart speaker and a Google Assistant-enabled phone.

#### Participants

Based on the Many Labs replication effect sizes for the five between-subjects experiments (reported below) and the intention to compare two media, when considering alpha = .05 and 1 – beta = .95, the minimum required sample size was assumed to be 200. The data collection occurred in January 2020. A total of 211 Americans were recruited using various social media, such as Facebook groups and Reddit channels related to virtual assistants and the crowdsourcing platform Prolific (Palan & Schitter, 2018). The ages of the participants ranged from 18 to 72 years (*M* = 34.27, *SD* = 11.34); 49.8% were male and 47.9% were female. Altogether, 111 persons used a smart speaker, and 100 persons participated via smartphone (of those, 33 used the keyboard, 62 their voice, and five a mix of the two to interact with the virtual assistant). All participants received a fixed compensation of 1.00 USD after completion. On average, the smart speaker users needed 4 min and 16.1 s (*SD* = 38.6 s) to complete the experiments. For the smartphone users, the completion time depended on how the answers were given: The participants who only used their keyboard (and probably often read the instructions) needed *M* = 2 min and 49.2 s (*SD* = 32.9 s). For those who used their voice (and probably often listened to the instructions), completion required *M* = 4 min and 0.4 (*SD* = 34.5 s). All reported times exclude attempts at setting up Survey Mate before the first use.

#### Materials and measures

We used the materials from the Many Labs 1 and 2 projects, with some minor changes. Full materials and detailed experimental descriptions, including original and Many Labs results, are available in the online supplement.

##### Allow/forbid experiment (Rugg, 1941)

In this experiment, the participants were assigned to one of two conditions. In one condition, the participants were asked whether the United States should forbid public speeches against democracy, while in the other condition, they were asked the opposite question, i.e., whether public speeches should be allowed. While opposite answers were expected, an asymmetry was found; 62% of the respondents in the forbid condition indicated “no,” while only 46% in the allow condition indicated “yes.” The results were replicated in the Many Labs 1 project (Klein, 2019; Klein et al., 2014) for US participants as well as on an international level.

##### Anchoring experiment (Jacowitz & Kahneman, 1995)

In this experiment, the participants were asked to estimate a set of numbers, such as the height of Mt. Everest or the number of babies born in the United States each day. Before estimating, they received an anchor value that was either too large (e.g., 50,000 babies; condition 1) or too small (e.g., 100 babies; condition 2). The comparison of estimates revealed a bias toward the anchor value (Jacowitz & Kahneman, 1995). This effect was replicated by Many Labs 1 (Klein et al., 2014) for US participants and on the international level. For brevity, in our reproduced experiment, the participants only had to estimate the number of babies.

##### Double-effect experiment (Hauser et al., 2007)

Hauser et al. (2007) compared participants’ willingness to kill a man in order to save five other people between a foreseen side-effects scenario and a greater good scenario. In the foreseen side-effects scenario, 89% of the participants considered it permissible to change the trajectory of a train so that it would kill one instead of five, but only 11% agreed to push a man in front of a train to achieve the same result in the greater good scenario. Many Labs 2 (Klein et al., 2018) replicated this difference between US participants and an international sample. In our reproduced experiment, the description of the greater good scenario was shortened slightly to enable it to be fully displayed to smartphone users.

##### Framing experiment (Tversky & Kahneman, 1981)

In this experiment, the participants considered a scenario about being in a store to buy a cheap or expensive item. In one condition, the participants were told that the cheap item was sold in another branch for 5 USD less. In the other condition, the expensive item was 5 USD less in that branch. When the participants were asked whether they would go to the other branch, 68% of those in the cheap item condition agreed to do so, but only 29% in the expensive item condition intended to go. This effect was replicated by Many Labs 2 (Klein et al., 2018) for US respondents as well as on the international level.

##### Less is better experiment (Hsee, 1998)

In this experiment, the participants were assigned to one of two conditions. They were asked to imagine that they had received a goodbye gift from a friend and to rate the generosity on a seven-point scale. In one condition, the gift was a coat purchased for 55 USD from a store where coats were sold for 50 USD to 500 USD. In the other condition, the gift was a scarf purchased for 45 USD from a store where scarfs ranged between 5 USD and 50 USD. The participants in the scarf condition considered the gift more generous despite its cost being less than that of the coat. The significant difference was also obtained by Many Labs 2 for both the US and international samples. In our reproduced experiment, generosity had to be rated on a ten-point scale ranging from 1 (not generous at all) to 10 (extremely generous) since it was expected to be easier to use than the original seven-point scale (which had the same poles), especially for participants who used a smart speaker. However, this adaption should have no impact on the reliability of the measurement (Cicchetti et al., 1985).

### Results

#### Reproduction of experiments

**Fig. 3:**
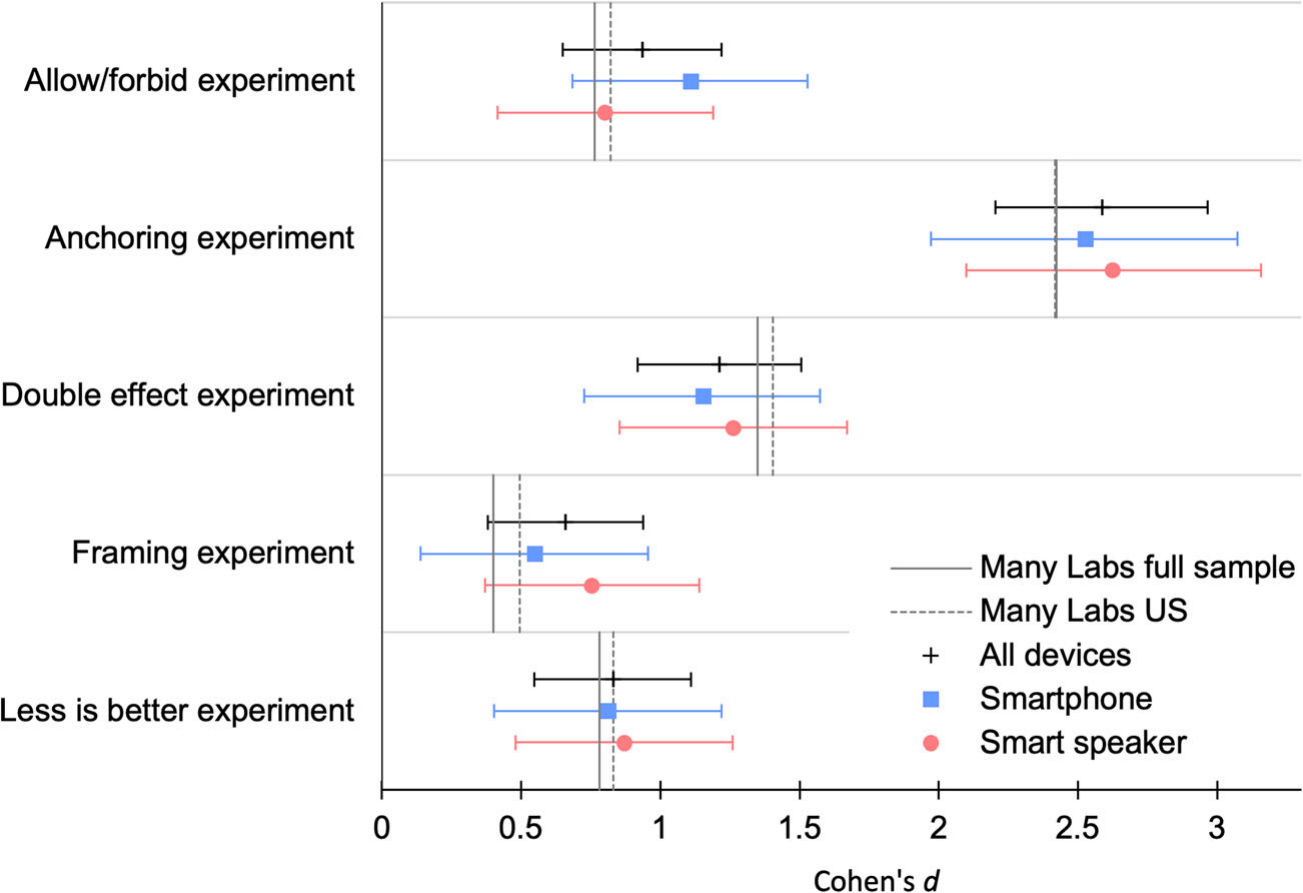
Effect sizes in the reproduced experiments. *Note:* Cohen’s *d* effect sizes and 95% confidence intervals for the reproduced experiments and the Many Labs replication results are depicted for comparison. All effects were significant at the 95% level and comparable for participants who used a smartphone or smart speaker

Data analysis was conducted in R. For all five experiments, we reproduced the analyses from the Many Labs 1 and 2 studies (Klein, 2019; Klein et al., 2014, 2018). All the effects were found to be of similar size. As shown in Fig. 3, the results were comparable between the smartphone and smart speaker users. However, the equivalence of the two media could not be assumed according to the non-significant TOST (Lakens, 2017) equivalence tests (see online supplement). For the smartphone users, no significant differences were found between the input modes (keyboard vs. voice). The analysis script and detailed results are provided in the online supplement.

#### Usability

As an indicator of usability, we investigated how often the instructions had to be repeated for the five experiments before an answer was collected. On average, 7.5% of the instructions were replayed because either the participants had requested the repetition, or the virtual assistant could not fully understand an answer. The number of repetitions varied by experiment, ranging from 5.2% in the framing experiment to 10.9% in the double-effect experiment. Since the scenario descriptions of the latter were much longer than those of other experiments (113 or 122 words depending on the double-experiment condition vs. 12 words in the allow/forbid experiment, 57 words in the framing experiment, 62 words in the anchoring experiment, and 67 words in the less is better experiment), we explored the effects of (a) instruction word count for each experiment (i.e., the number of words of the scenario description and question), (b) device type, and (c) their interaction in terms of the number of repetitions in the experiments. The respective linear regression revealed a marginally significant effect of word count, *β* = 0.00075, 95% CI = [– 0.000066, 0.0016], *p* = .071. Neither the type of device nor the interaction seemed to play a role, both *|t|* < 1.

### Discussion

Five classic and contemporary psychological experiments were successfully reproduced using data collected by a virtual assistant on both smartphones and smart speakers. The effects were comparable to those obtained in the Many Labs 1 and 2 replication studies (Klein et al., 2014, 2018) and appeared similar for the different data collection devices. However, there may have been differences between smartphone users using different input and output modes. As it was not possible to detect whether they had read or listened to instructions due to technical limitations, and the sample size was too small to detect differences between the written and spoken answers, further research should focus on the benefits and limitations of different input and output modes. We subsume that current smartphone and smart speaker technology are capable of collecting valid results in scenario-based experiments (R3), although the length of the scenario may play a role. While the effect was not significant, the instruction word count may relate to more errors and repetitions for longer experiments than those applied here. Thus, working with longer scenarios might have been more difficult for participants, likely hampering the usability of the medium and limiting the quality of the collected data.

## General discussion

The results of the two studies indicate that virtual assistants are suitable for the collection of high-quality data. The first study revealed that indicators of reliability and validity are comparable (not necessarily equivalent) for data assessed by Survey Mate and an online questionnaire. For both media, the internal consistencies were high, and the correlations with other scales and behavior intentions in the two fictitious scenarios signaled construct and criterion validity. The mean values were also comparable for most scales. However, the assistant users showed a higher exaggeration of the positive qualities assessed by the KSE-G instrument, which suggests stronger respondent social desirability bias. This finding is consistent with that of previous research showing that sensitive information is less accurately reported when using interactive voice response systems instead of online questionnaires (Kreuter et al., 2008). The reason may be stronger fears among virtual assistant users about the potential repercussions of disclosing sensitive information (Tourangeau & Yan, 2007), an assumption that also bolsters the finding that the assistant users had significantly lower trust in the protection of their data compared to participants who completed the online questionnaires. While the original statement phrasing of the scales could be employed to assess reliable and valid data, the participants reported greater enjoyment about answering questions. Rephrasing scale items into questions may support the more natural style of conversations between the virtual assistant and the user (Kamm, 1995; Moore & Arar, 2019), likely affecting the acceptance of and participation in virtual assistant surveys. In the second study, we utilized Survey Mate to reproduce five experiments from social psychology that had been successfully replicated by the Many Labs 1 and 2 projects (Klein et al., 2014, 2018). Comparable effects were observed, indicating the suitability of virtual assistants in conducting scenario-based experiments. However, the results suggested that longer scenarios may be related to increased errors. Depending on the device, virtual assistant users are not always able to read a scenario multiple times before responding. Thus, errors and repetitions are inevitable when presenting long and complex scenario descriptions. In general, the time needed to complete the study played a crucial role. Data from the first study show that completing the survey with the virtual assistant was more time-consuming than answering the comparable online questionnaire. While the latter could present multiple items simultaneously, the linear conversation with a virtual assistant decelerated item presentation and response collection. Furthermore, for the virtual assistant users, the duration from survey start to finish varied by device. In the second study, the participants were considerably faster when using a smartphone keyboard instead of their voice. When using the keyboard, Google Assistant automatically stopped reading out instructions when the user was looking at the screen. As reading the instructions was less time-consuming than listening to them, the survey could be completed in less time. Studies conducted with a virtual assistant should comprise a limited set of items, not only because participation requires more time but also because the medium is designed for short-term interactions. People use virtual assistants to check the weather or play a short quiz while waiting for the bus or preparing dinner; 20-min surveys do not fit this usage pattern. While the optimal length of a survey should be assessed in future research, the studies presented here show that virtual assistants are capable of collecting multiple scales or running five short experiments in approximately 5 min. A combination of the two should also be possible. Based on our findings, we can infer a set of recommendations for the use of voice assistants in survey or experimental research (see Textbox 1).

### Applications

Virtual assistants should not be considered a mere replacement for established data collection tools, such as telephone interviews or online questionnaires. They may allow the recruitment of participants who are usually reluctant to participate, including the blind and people with dyslexia or lower levels of education who do not volunteer for lengthy questionnaires that involve a considerable amount of text. Because of the widespread use of virtual assistants, they should be considered an important tool for behavioral insights research. The possibility of drawing results from diverse populations could help inform policymaking and improve public services. For instance, virtual assistants could be an effective tool to survey, in real time, feelings, risk perceptions, and behaviors during the COVID-19 pandemic. Furthermore, virtual assistants should be considered an inexpensive data collection tool for low- and middle-income countries. For instance, in many African countries, people do not own a computer but a smartphone (Pew Research Center, 2019), and surveys could be rolled out in multiple languages using the often preinstalled Google Assistant. Behavioral scientists routinely draw broad claims from Western, educated, industrialized, rich, and democratic (WEIRD) samples. Often, their findings cannot be generalized to other populations (Henrich et al., 2010). Virtual assistants could help in collecting data from samples representing other populations, rendering them an important methodological contribution to behavioral science.

### Limitations

Our findings should be seen as a starting point for future work on the benefits and limitations of the medium. While the initial results are promising, researchers should consider the following issues when employing virtual assistants for data collection. Primacy and recency effects known from paper and online questionnaires should be considered in the context of virtual assistants; likewise, the effects of assistant voice characteristics on answers and perception of the medium should be investigated. Additional work is also required to assess the suitability of virtual assistants for collecting data for different domains and scales, especially when questions relate to sensitive issues where data protection may be especially relevant. It is worth exploring whether different interaction patterns and voice styles affect the humanness of the virtual assistant and, thus, influence trust.

There is a high chance that the participants recruited for both studies were frequent users of virtual assistants and had fewer privacy concerns than the average US citizen. Consequently, recruiting participants from a representative sample could be difficult. The lower trust in the data protection of virtual assistant-based assessments compared to established media-based assessments could threaten widespread adoption. Future research is necessary to evaluate the acceptance of psychological surveys and experiments among occasional users of virtual assistants.

Furthermore, recruitment strategies need to be elaborated. Ideally, participants can set up a tool such as Survey Mate and keep returning for new surveys. Of course, this would require some kind of incentivization. Money could be a suitable motivator. Moreover, psychology often creates a high level of lay interest. Thus, easily understood background information about the studies in which people participated could also be an appropriate reward. For example, in the first study on assessing procrastination, the participants could receive feedback about their score, indicating how much they procrastinate compared to other participants or the population average; tips on how to overcome procrastination could also be provided. In the future, different incentives should be evaluated and compared.

The applicability of virtual assistants for research outside WEIRD countries should also be examined. In low- and middle-income countries, acceptance and usability criteria may differ, requiring adaptions of tools such as Survey Mate. Furthermore, participation requires at least a smartphone and internet connection, making it difficult to reach those without access to the technology, e.g., low-income groups in developing countries.

**Textbox 1 Recommendations**

**Table:** 

Taking into account advantages and challenges, we derive the following preliminary recommendations for the use of voice assistants in survey or experimental research: **(1) Ensure brevity** Since participating in a virtual assistant-powered survey consumes more time compared to completing a questionnaire, surveys should be as short as possible. Scenario descriptions, instructions, and questions should be reduced to a minimum. They must also be easy to understand when heard for the first time—cognitive pretesting is essential. Otherwise, participants’ attention may decrease, potentially deteriorating data quality and increasing the number of dropouts. In this regard, the same precautions applied to the development of telephone interviews (Hansen, 2006) should be considered when designing virtual assistant-based surveys. **(2) Use items that support conversational flow** Many validated scales assess agreement with statements using a set of predefined answers. Assessing these scales with a virtual assistant can produce valid data. However, the participants in our first study reported significantly more enjoyment when statements were rephrased into questions (data quality did not change). Consequently, before using standard scales, their wording should be checked, and adaptation to a question–answer format should be considered in order to support a more natural style of conversation between the virtual assistant and the user (Kamm, 1995; Moore & Arar, 2019). **(3) Consider social desirability bias** Virtual assistants may be limited in terms of accurate assessment of very sensitive information and should not be employed for research that is prone to social desirability effects. However, if information about sensitive topics has to be assessed, questionnaire design techniques that are known to mitigate bias (Nederhof, 1985) may be adapted for virtual assistants. While they have not been validated, methods such as the normative technique, i.e., asking participants about the behaviors of close friends instead of their own (Yeatman & Trinitapoli, 2011), could be considered when creating surveys for virtual assistants. Furthermore, the short version of a social desirability scale could be included to assess desirability effects. **(4) Ensure and communicate data privacy** Previous research on virtual assistants did not reveal a strong relationship between data privacy concerns and usage frequency (Dubiel et al., 2018). However, privacy concerns may affect response styles, increase social desirability bias, and curtail data quality. Similar to other research, at the beginning of each survey, participants should be informed about how their data are collected, processed, stored, and employed as well as which third parties have access. Thus, using voice assistants requires additional attention to explain how data are protected.	

## Conclusions

While social desirability, privacy concerns, and the limited duration of data collection could be identified as constraints, virtual assistants should be considered a promising medium for psychological and behavioral research. They are available to billions of users, easily accessible, and suitable for the collection of high-quality data in both survey and experimental research. The application of virtual assistants is not limited to geographic regions; languages are easily adaptable; and these assistants allow the recruitment of samples that are not usually recruited by established data collection techniques.

We would like to encourage researchers to further evaluate and use this new medium, either by developing their own extensions for established assistants or through Survey Mate collaborations. Exploring the opportunities offered by this new medium can improve the generalizability of theories and yield an important building block for multi-method research in various contexts. We are confident that future innovations in the field of human-computer interaction will further improve the capability of virtual assistants for data collection tasks.

## Declarations

Both studies were conducted in accordance with German Psychological Association guidelines. All participants provided informed consent to use and share their data for scientific purposes without disclosure of their identities. The research was conducted at a German university, where institutional review boards or committees are not mandatory. It is negligible-risk research, with no foreseeable risk of harm or discomfort other than potential inconvenience expected from participation. All participants knew that they were free to quit the study at any time.
